# Effects of omega-3 polyunsaturated fatty acids on cellular development in human ovarian granulosa tumor cells (KGN)

**DOI:** 10.3389/fnut.2022.1017072

**Published:** 2022-09-30

**Authors:** Yilin Yao, Shen Tian, Ningxin Li, Yanzhou Yang, Cheng Zhang

**Affiliations:** ^1^College of Life Science, Capital Normal University, Beijing, China; ^2^Key Laboratory of Fertility Preservation and Maintenance, Key Laboratory of Reproduction and Genetics in Ningxia, Ministry of Education, Department of Histology and Embryology, Ningxia Medical University, Ningxia, China

**Keywords:** PUFAs, ovarian cancer, KGN cells, GLUT, OCT4

## Abstract

Emerging research has shown that polyunsaturated fatty acids (PUFAs) benefit human health and exert anti-cancer effects. However, there is little understanding of the specific mechanisms by which PUFAs regulate the cells of the ovarian granulosa tumor. In the current study, we investigate the effects and the possible mechanisms of PUFAs on human ovarian tumor cells development. KGN cells were treated with omega-3. Small interfering (siRNA) and specific activator were used to knock down and overexpress gene expression in KGN cells. The protein content levels were analyzed by Western blot. Cell viability, proliferation and apoptosis assay were performed to examine the cellular development. And the level of glucose uptake in KGN cells were assessed by 2-DG measurement. The results showed that omega-3 treatment reduced cell viability, proliferation and increased cell apoptosis. Further studies showed that omega-3 also reduced GLUT1/4 protein content and cellular glucose uptake. Subsequent knockdown and overexpression of OCT4 using *Oct4* siRNA and O4I2 (OCT4 activator) showed that OCT4 was involved in the regulations of omega-3 on GLUT1/4 expression and cell development. Our data demonstrate that omega-3 inhibits cellular development by down-regulating GLUT1/4 expression and glucose uptake in KGN cells, which are mediated through OCT4.

## Introduction

It is well known that ovarian cancer is one of three types of malignant tumors of the female reproductive system, which is only after cervical cancer and endometrial cancer (1, 2). Ovarian cancer often occurs in perimenopausal women. However, the occurrence of ovarian cancer is increased and has the trend of younger in recent years in females of the world (3). Usually, the patient with ovarian cancer is often asymptomatic in the early period. Once the patient is diagnosed, 60–70% of patients are already in phase III-IV or with abdominal metastasis. Moreover, the 5-year survival rate is only around 30% although the treatments such as surgery, radiotherapy, or chemotherapy are taken (4).

It has been reported that ovarian cancer is a kind of estrogen-dependent cancers. Within the category of sex cord-stromal tumors, ovarian granulosa cell tumor (GCT) is the most commonly diagnosed type. GCT has several characteristics, including ovarian enlargement, high estrogen levels, recurrence rate, and the possibility of malignancy and metastasis (5). And these estrogen-dependent cancers can also be inhibited by anti-estrogens (6). It is well known that the aromatase enzyme which found in granulosa cells, irreversibly catalyzes the conversion of androgens into estrogen for follicular growth and ovulatory coordination. Furthermore, estrogen may act through a homeodomain transcription factor, Octamer-binding transcription factor 4 (OCT4) known as a member of the Pit-Oct-Unc (POU) family of transcription factors. Our previous study showed that OCT4 is related to the granulosa cell growth, which is regulated by estrogen receptor β (ERβ) and P450 lanosterol 14a-demethylase (CYP51) (7).

An increasing number of research suggests that certain ingredients in food may protect against cancer or prevent it from becoming cancerous (8). Several reports and clinical researches have indicated that PUFAs (Polyunsaturated fatty acids) are important for female reproduction. The rational ratio of omega-3/omega-6 PUFA improves the expression of steroidogenesis enzymes which are involved in hormone synthesis and ovarian functions (9–11). Lower plasma cholesterol level is associated with higher level of omega-3 in diets (11). Omega-3 is one of PUFAs, which are 16–22 carbon atoms in a carbon chain of PUFAs and straight chain fatty acids with two or more double bonds. Abundant supply of omega-3 PUFAs are found in fish oil and linseed oil, such as eicosapentaenoic acid (EPA) and docosahexaenoic acid (DHA) (12, 13).

There is growing evidence that increased intake of PUFAs can have positive impact on human health (14–16). Many studies have shown that PUFAs has positive effects on the treatment of colon, breast, and prostate cancer by causing cell death or cancer prevention (17–24). And it has been reported that PUFAs as supplements enhances the effect of chemotherapy on cancer (20). Moreover, DHA also inhibits the cellular proliferation, cycle arrest, and apoptosis in Hey and IGROV-1 cells derived from ovarian cancer patient (25). Although a number of studies have reported that PUFAs treat and prevent cancer through a variety of mechanisms, whether and how omega-3 PUFAs affect ovarian GCTs still remains uncertain.

It is well known that substantial energy is necessary for the cell growth, and glucose appears to be the dominant energy substrate (26, 27). Unlike most normal tissues, many tumor cells depend on aerobic glycolysis even when oxygen is sufficient to support mitochondrial oxidative phosphorylation, a phenomenon known as the Warburg effect (28, 29). Glucose is transferred by glucose transporter proteins (GLUTs) since it cannot penetrate the cell membrane. GLUTs are carrier proteins embedded in cell membranes, widely distributed in various tissues, and affected by metabolism and hormones (30–32). It has been reported that many cancer types are related to up-regulated GLUTs expression as a result of perturbation of gene expression or protein relocalization or stabilization (33). GLUTs are also involved in the regulation of ovarian cancer cells (34). Additionally, ciglitazone inhibits GLUT1 to increase the death of ovarian cancer cells (35). Moreover, fish oil reduced GLUT4 protein levels in skeletal muscle of diabetic rats (36). Although omega-3 plays important roles in regulating ovarian function and treating or preventing cancer, the exact mechanism of its action on ovarian GCTs remains unclear.

Our study aimed to investigated the possible mechanisms that omega-3 regulates KGN cells development. We demonstrated that omega-3 decreases GLUT1/4 expression and inhibits cell development, which are associated with decreased OCT4 expression.

## Materials and methods

### Reagents and antibodies

Unless otherwise noted, all reagents used in current study were purchased from Sigma Chemical Co. (St. Louis, MO, USA). DMEM/F-12 and Trypsin were purchased from Biological Industries (Beit Haemek, Israel). Fetal bovine serum was from PAN-Biotech (Bavaria, Aidenbach, Germany). The products of Antibiotic-Antimycotic (100x) and Lipofectamine 3000 Transfection Reagent were from Invitrogen (Carlsbad, CA, USA). The inducer of OCT3/4 (O4I2) was provided by Selleck Chemicals (Houston, TX, USA). Rabbit polyclonal anti-OCT4 (ab19857), rabbit polyclonal anti-GLUT4 (ab33780), and rabbit polyclonal anti-GAPDH (ab9485) were purchased from Abcam (Cambridge, MA, USA). Anti-Glucose Transporter GLUT1 (#12939) rabbit monoclonal antibody was obtained from Cell Signaling Technology, Inc. (Beverly, MA, USA). Horseradish peroxidase (HRP)-conjugated anti-rabbit and anti-goat IgG were from Santa Cruz Biotechnology, Inc. (Santa Cruz, Beijing). The enhanced chemiluminescence (ECL) detection kit was obtained Amersham Life Science (Oakville, ON, Canada). Acrylamide (electrophoresis grade), N, N′-methylene-bis-acrylamide, ammonium persulfate, glycine, and SDS-PAGE prestained molecular weight standards were purchased from Bio-Rad (Richmond, CA, USA). The one-step terminal deoxynucleotidyl transferase 2′-deoxyuridine, 5′-triphosphate nick-end labeling (TUNEL) cell apoptosis detection kit was from KeyGEN (Beijing, China). BeyoClickEdU Cell Proliferation Kit with Alexa Flour 488 was purchased from Beyotime Biotechnology (Shanghai, China).

### Human granulosa-like tumor cell line

A humidified atmosphere of 5% CO_2_/95% O_2_ at 37°C was used for the maintenance of human ovarian granulosa-like KGN cells in DMEM/F-12 medium supplemented with 10% (vol/vol) fetal bovine serum and 1% antibiotic-antifungal. Culture medium was replaced with serum-free medium for 12 h as starvation treatment. Then, cells were treated with DHA (25 μM) (37, 38) or O4I2 (12.5 μM) (39) according to the experimental requirements.

### RNA interference

KGN cells were transfected with small interferences (siRNA) in 6-well plates at 70–90% confluence according to the manufacturer’s protocol. In brief, *Oct4* siRNA (JTS Scientific) and scrambled sequence control (JTS Scientific) solution was added to serum-free DMEM, gently mixed, and incubated for 5 min at room temperature. Lipofectamine 3000 (Invitrogen) was then added to the above solution and immediately thoroughly mixed and left at room temperature for 10 min to allow complex formation. Finally, the complex was added drop by drop to the wells containing KGN cells and medium. The cells were transfected with *Oct4* siRNA and scrambled sequence control for 48 h with Lipofectamine 3000 according to the manufacturer’s instructions.

### Protein extraction and western blot

Western blot analysis was performed as described previously (40). Briefly, the cells were lysed with lysis buffer [30 mM NaCl, 0.5% Triton X-100, 50 mM Tris-HCl (pH 7.4) with a protease cocktail and phosphatase inhibitor (Sigma-Aldrich, MO)] for 30 min at 0°C. The supernatant was collected after the insoluble fractions were removed by centrifugation (15,000 × g, 4°C, 30 min). And then, the protein concentration was determined with the BCA Protein Assay Kit (Beyotime Biotechnology, Shanghai, China) according to the manufacturer’s instructions. For each sample, 20 μg (depending on the individual experiments) of total proteins were separated on 10% sodium dodecyl sulfate-polyacrylamide gel electrophoresis (SDS-PAGE) and electrically transferred to polyvinylidene difluoride (PVDF) membranes (Roche, Basel, Switzerland). The membranes were then blocked in PBST buffer [PBS (10 mM phosphate, 150 mM NaCl, pH 7.4) containing 0.05% Tween-20] containing 5% bovine serum albumin at room temperature for 1 h then incubated (4°C, overnight) with diluted primary antibody [polyclonal anti-OCT4 (1:1,000), polyclonal anti-GLUT1 (1:1,000), polyclonal anti-GLUT4 (1:1,000), or monoclonal anti-GAPDH (1:5,000)], followed by HRP-conjugated secondary antibody (1:2,000–1:5,000; 2 h, RT). Peroxidase activity was visualized using SuperSignal West Pico Chemiluminescent Substrate (Thermo Fisher Scientific, USA) with an ImageQuant LAS 4000 Mini Imaging System (Cytiva, USA) according to the manufacturer’s instructions. Immune response signals were analyzed using AlphaEaseFC 4.0 (Alpha Innotech, USA).

### Glucose uptake assay

Following the manufacturer’s protocol, glucose uptake levels were determined using a commercial kit (Cosmo Bio Co., Ltd., Tokyo, Japan). This system is based on an enzymatic photometric test method to directly measure the amount of 2-deoxyglucose-6-phosphate (2DG6P), which monitors glucose uptake by detecting the nicotinamide adenine dinucleotide phosphate generated during the oxidation of 2DG6P to 6 –phospho-2-deoxyglucuronic acid. Briefly, KGN cells were cultured as mentioned above. For measurement, the medium was removed from the culture plate wells, and the cells were incubated for 6 h in a serum-free medium. After washing, the cells were incubated with 2DG solution for 20 min at 37°C. Medium was removed and the cells were gently rewashed three times before being disrupted by the microtip sonicator. And the lysate was collected and heated at 80°C for 15 min. Then, the samples were centrifuged (4°C, 15,000 × g) for 20 min. The OD of the wells was read at 420 nm using a microplate reader. The concentration of each sample was measured in triplicate and calculated using the standard curve provided in the kit.

### Cell viability analysis

Cell Counting Kit-8 (Dojindo, Kumamoto, Japan) was used to detect the viability of cells (41). KGN cells were seeded in 96-well plates and processed as required. Each well was then filled with CCK-8 solution (10 μL) and incubated for 2 h at 37°C. And then, microplate reader was used to determine optical density (OD) at 450 nm. The mean OD value of each treatment was used as an indicator of cell viability.

### EdU incorporation assay

According to the manufacturer’s instructions, EdU cell proliferation assays were conducted. In brief, upon treatment, KGN cells were incubated for 2 h with 50 μL EdU. After 30 min of paraformaldehyde fixation at room temperature, the cells were permeabilized in 0.5% Triton X-100 for 10 min. In order to detect cell nuclei, EdU staining was performed, followed by Hoechst 33342 counterstaining for 30 min. Nuclei positive for EdU were detected using a Laser Scanning Microscope LSM 780 (ZEISS, Jena, Germany). Nucleated cells incorporating EdU in five high-power fields per well were used to calculate cell proliferation rates.

### Triphosphate nick-end labeling apoptosis assay

According to manufacturer’s instructions, apoptotic cells were identified by using a commercial reagent kit One-Step TUNEL Apoptosis Assay Kit (Beyotime Biotechnology, Beijing, China). Briefly, PBS wash was followed by 30 min of 4% paraformaldehyde fixation at room temperature. Then, KGN cells were permeated in 1% Triton X-100 at room temperature about 2 min. Following PBS wash, each sample was added the TdT enzyme reaction and placed into the wet box at 37°C incubator for 1 h. The negative samples without the TdT enzyme reaction liquid. Streptavidin-TRITC labeling buffer was dropped onto the cells which in a wet box, and placed at 37°C for 30 min to avoid light. Finally, cells were incubated with DAPI staining solution at room temperature for 10 min to detecting nuclei. After washing by a buffered PBS solution, Zeiss ZEN lite software was used to record the images of the cells taken with a Laser Scanning Microscope LSM 780 (Zeiss, Oberkochen, Germany).

### Statistical analysis

Experimental procedures were repeated at least three times in each group, and the results were presented as means ± SEM, as detailed in the figure legends. *T*-tests or one-way ANOVA were used to compare treatments statistically, and Bonferroni post-tests were used to compare means when significant differences were detected with GraphPad Prism 8.0 statistical software (GraphPad Software, Inc., San Diego, CA). It was considered statistically significant when *P* < 0.05.

## Results

### Effect of omega-3 on cellular development in KGN cells

**FIGURE 1:**
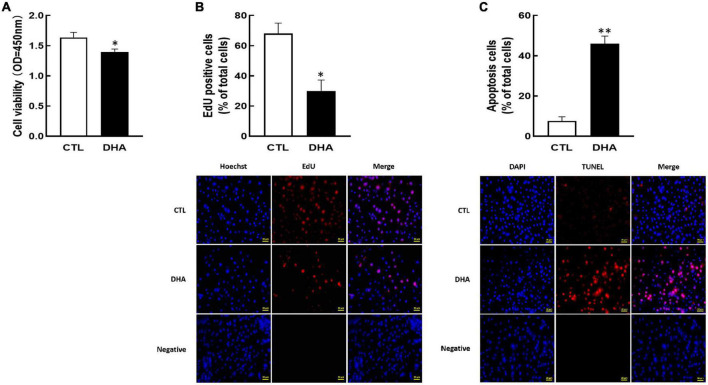
Effect of Omega-3 on cellular development in KGN cells. The cells were cultured for 24 h in the absence or presence of omega-3 (25 μM), and then cell viability **(A)**, proliferation **(B)** and apoptosis **(C)** were detected by CCK-8 assay, EdU and TUNEL measurement, respectively. Data are presented as mean ± SEM of three independent experiments. *P < 0.05, ^**^P < 0.01 compared with control (CTL). Bar = 50 μm.

To demonstrate the effect of omega-3 on KGN cell growth, the cell development and apoptosis were investigated after omega-3 treatment. As shown in Figure 1, omega-3 significantly reduced cellular viability (*P* < 0.05, Figure 1A) and proliferation (*P* < 0.05, Figure 1B). And the number of apoptosis cells was markedly increased by omega-3 (*P* < 0.01, Figure 1C).

### Effect of omega-3 on GLUT1/4 expression in KGN cells

**FIGURE 2:**
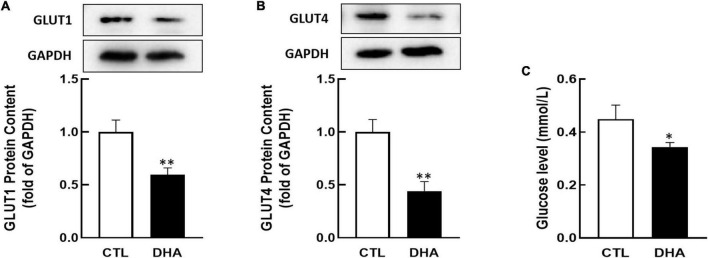
Effect of Omega-3 on GLUT1/4 expression and glucose uptake in KGN cells. KGN cells were cultured with omega-3 (25 μM) for 24 h. The proteins level of GLUT1 **(A)** and GLUT4 **(B)** were assessed by Western blot analysis. And the cellular glucose uptake **(C)** was assessed by 2-DG measurement. Data are presented as mean ± SEM of three independent experiments. *P < 0.05, ^**^P < 0.01 compared with CTL.

Researchers have found that GLUT1 and GLUT4 play important roles in ovarian cell growth (40–42). KGN cells were cultured with omega-3 and the proteins content of GLUT1/4 were analyzed by Western blot. In Figure 2, the results showed that omega-3 decreased both of GLUT1 and GLUT4 proteins expression (*P* < 0.01, Figures 2A,B).

Glucose is critical to the cell growth. To determine if omega-3 regulates glucose uptake, KGN cells were incubated with omega-3 for 24 h and cellular glucose were determined by 2-DG. As shown in Figure 2C, the glucose uptake in cells was significantly decreased by omega-3 (*P* < 0.05).

### Effects of OCT4 on cellular development

**FIGURE 3:**
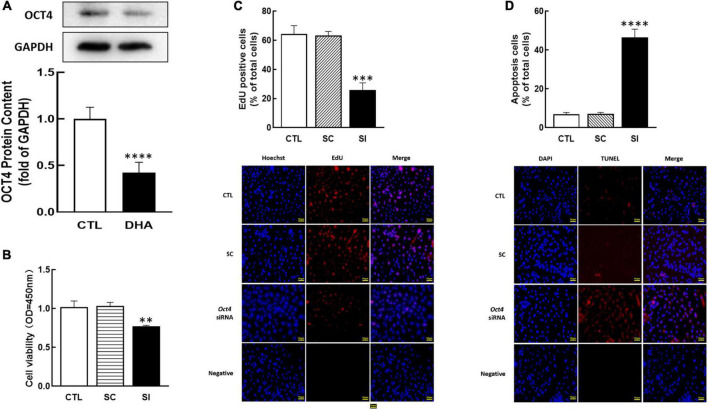
Effect of Omega-3 on OCT4 expression and the effect of *Oct4* siRNA on KGN cells development. The cells were treated with omega-3, and OCT4 protein content was detected by Western blot **(A)**. After transfected with *Oct4* small interfering (SI) RNA (scrambled sequence as control, SC) for 48 h using Lipofectamine 3000, the cell viability **(B)**, proliferation **(C)**, and apoptosis **(D)** were analyzed by CCK-8 assay, EdU and TUNEL measurement, respectively. Data are presented as mean ± SEM of three independent experiments. ^**^P < 0.01, ^***^P < 0.001, ^****^P < 0.0001 compared with CTL. Bar = 50 μm.

As a member of the POU family, OCT4 is also essential in female reproduction. To study the effect of omega-3 on OCT4 expression, cells were treated with omega-3 for 24 h. Results showed that omega-3 significantly reduced OCT4 expression compared to control (*P* < 0.0001, Figure 3A).

To investigate the role of OCT4 on cell development, OCT4 expression was knocked down by siRNA in KGN. The results showed that *Oct4* knockdown significantly reduced cell viability (*P* < 0.01, Figure 3B), decreased the number of proliferative cells (*P* < 0.001, Figure 3C), and significantly increased cell apoptosis (*P* < 0.0001, Figure 3D).

**FIGURE 4:**
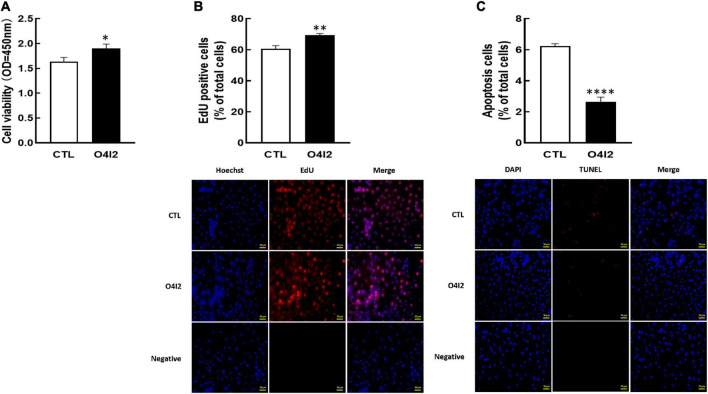
Effect of OCT4 activator (O4I2) on cellular development in KGN cells. The cells were treated with O4I2 (12.5 μM) for 24 h and then CCK-8 assay, EdU and TUNEL measurement were used for detecting cell viability **(A)**, proliferation **(B)**, and apoptosis **(C)**. Data are presented as mean ± SEM of three independent experiments. *P < 0.05, ^**^P < 0.01, ^****^P < 0.0001 compared with CTL. Bar = 50 μm.

For further determine the functions of OCT4 in KGN cells, we cultured cells with OCT4 activator (O4I2). The results revealed that O4I2 treatment increased cell viability (*P* < 0.05, Figure 4A) and promoted cell proliferation (*P* < 0.01, Figure 4B) compared those with the control group, respectively. The TUNEL results showed that O4I2 significantly inhibited cell apoptosis (*P* < 0.0001, Figure 4C).

### Effects of OCT4 on cellular GLUT1/4 expression and glucose uptake

**FIGURE 5:**
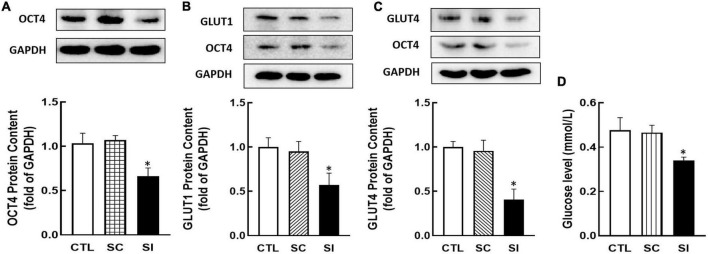
Effect of *Oct4* siRNA on GLUT1/4 expression and glucose uptake in KGN cells. The protein contents of OCT4 **(A)**, GLUT1 **(B)**, GLUT4 **(C)**, and glucose uptake **(D)** were detected by Western blot analysis or by 2-DG measurement after the cells were transfected with *Oct4* siRNA (100 nM) for 48 h. Data are presented as mean ± SEM of three independent experiments. *P < 0.05 compared with CTL.

To investigate whether OCT4 regulates GLUT1/4 expression, KGN cells were transfected with *Oct4* siRNA. As shown in Figure 5, knockdown of *Oct4* (*P* < 0.05, Figure 5A) significantly decreased GLUT1 and GLUT4 expression (*P* < 0.05, Figures 5B,C). Meanwhile, knockdown of OCT4 expression also reduced cellular glucose uptake (*P* < 0.05, Figure 5D).

**FIGURE 6:**
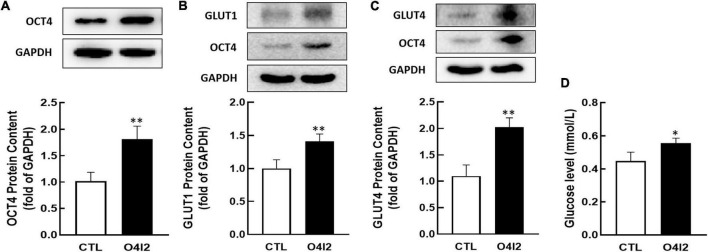
Effect of O4I2 on GLUT1/4 expression and cellular glucose uptake. KGN cell were co-cultured with O4I2 (12.5 μM) for 24 h. The protein contents of OCT4 **(A)**, GLUT1 **(B)**, and GLUT4 **(C)** content were assessed by Western blot analysis. And 2-DG measurement was used for detecting the cellular glucose uptake **(D)**. Data are presented as mean ± SEM of three independent experiments. *P < 0.05, ^**^P < 0.01 compared with CTL.

In addition, Western blot results showed that O4I2 significantly increased the expression of OCT4 (*P* < 0.01, Figure 6A), at the same time, GLUT1 and GLUT4 expression (*P* < 0.01, Figures 6B,C) were up-regulated too. The cellular glucose uptake was also detected after treated with O4I2. The results showed that O4I2 promoted KGN cells glucose uptake (*p* < 0.05, Figure 6D).

## Discussion

In several studies, it has been found that PUFAs have positive effects on different cancer treatments by causing cell death or cancer prevention. Previous studies have demonstrated the therapeutic effects of PUFAs on ovarian cancer cells and transgenic mice. For example, DHA significantly inhibited cellular proliferation, induced cell cycle arrest and caused apoptosis in Hey and IGROV-1 cells (25). In addition, PUFAs can induce apoptosis of human ovarian cancer cell line KF28 at physiological concentration levels through activation of ROS-dependent MAP kinase (43). And DHA can enhance the cisplatin cytotoxicity in ovarian cancer cells (44). However, the effects and mechanisms of PUFAs on GCT of ovary still remain unclear. We demonstrated that OCT4 is a novel target of omega-3 fatty acids in the present study, which regulates ovarian cancer cells growth. Our studies demonstrated that omega-3 decreased GLUT1/4 expression and cellular glucose uptake by down-regulating OCT4 expression, which in turn inhibited KGN cells development. It has been reported that PUFAs affect cellular membrane fluidity, signaling cascades, and oxidative damage susceptibility (45, 46). A number of studies have shown that PUFAs inhibits different types of cancer cells (17–19, 21, 22, 24). For example, the development of non-small lung tumors can been significantly inhibited by the omega-3 polyunsaturated fatty acid DHA (37), and TGF-β-induced epithelial-to-mesenchymal transition (EMT) in human breast cancer cells is suppress by DHA (38), etc. In the present study, omega-3 dramatically decreased KGN cells development and increased cellular apoptosis as a positive effector. However, the mechanisms by which PUFAs regulates ovarian GCT are still unclear.

It is well known that ovarian cancer is one of the estrogen-dependent cancers. The human GCT-derived cell line KGN has been used to investigate GCT igenesis *in vitro* (47–49). It has been reported that PUFAs regulate steroidogenesis by directly affecting the enzymes such as steroid acute regulator (StAR) and cytochrome P450 about steroid synthesis (45, 50). Moreover, PUFAs are also involved in the regulations of reproductive endocrinology as the direct precursors of PGs (51–53). It is possible that omega-3 negatively regulates KGN cells growth by inhibiting estrogen synthesis since OCT4 expression was also down-regulated.

OCT4, as a member of the POU family, is detected in granulosa cells (54, 55). And OCT4 not only promotes the formation of stem cells and germ cells, but also increases granulosa cell development (56). In our previous study, the expression of OCT4 is regulated by ERβ and CYP51, which are related to the synthesis and effects of estrogen (7). In the present study, omega-3 down-regulated OCT4 expression. Meanwhile, knocking down OCT4 increased the percentage of cell apoptosis. Oppositely, OCT4 activator inhibited cellular apoptosis and promoted cellular growth. As a result of these findings, OCT4 is directly involved in the regulation of KGN cell growth. Interestingly, once the expression of OCT4 was changed, the expression of GLUTs were also changed in KGN cells. Meanwhile, the glucose uptake was also changed along with the changes of OCT4 expression. The present results provide further evidence that OCT4 possibly affects ovarian cancer cells growth not only by regulating steroidogenesis but also by changing substantial energy.

The hydrophilic molecule glucose cannot permeate the cell membrane, and its utilization requires the transport and catalysis of GLUTs, which is a protein family, including a variety of proteins, widely distributed in various tissues in the body (7, 30–32). In this study, we showed that omega-3 significantly down-regulated GLUT1 and GLUT4 proteins content. Decreased cellular glucose uptake via lower level of GLUTs may explain that omega-3 attenuates ovarian cancer cells growth. Interestingly, these changes are mediated by OCT4. These results provided new evidence that omega-3 directly or indirectly regulates estrogen synthesis, and the latter further regulates OCT4 expression (10, 57). Moreover, it is possible that other transcription factors may activate the *Oct4* promoter region, regulating the expression of GLUTs (58–60).

## Conclusion

**FIGURE 7:**
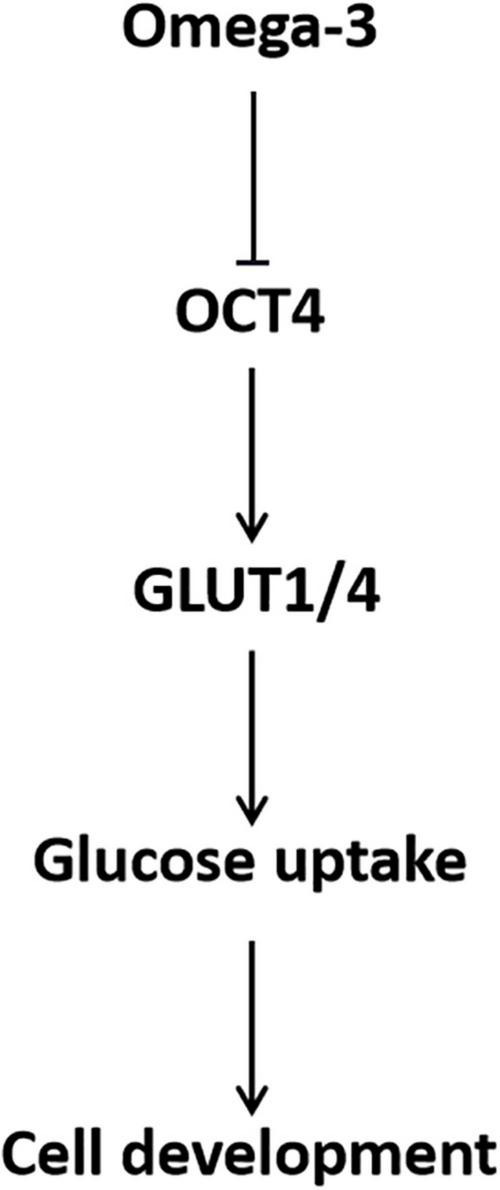
Schematic of the role of OCT4 in omega-3 inhibited cellular glucose uptake and development in KGN cells.

In conclusion, omega-3 decreased GLUT1/4 expression and cellular glucose uptake through down-regulation of OCT4, which in turn inhibited KGN cell development (Figure 7). The findings of this study provide a preclinical theoretical basis for the application of PUFAs in dietary intervention and adjuvant therapy for patients with ovarian GCT.

## Data availability statement

The raw data supporting the conclusions of this article will be made available by the authors, without undue reservation.

## Author contributions

CZ: conceptualization and funding acquisition. CZ and YZY: resources. YLY and NL: investigation. YLY: data curation. CZ and YLY: writing—original draft. CZ and ST: writing—review and editing. All authors have read and agreed to the published version of the manuscript.

## Abbreviations

PUFAs: Polyunsaturated fatty acids
DHA: Docosahexaenoic acid
GCT: Granulosa cell tumors
OCT4: Octamer-binding transcription factor 4
GLUT: Glucose transporter protein
CCK-8: Cell Counting Kit-8
GAPDH: Glyceraldehyde-3-phosphate dehydrogenase
OD: Optical density
DMEM/F-12: Dulbecco’s Modified Eagle Medium/Nutrient Mixture F-12
ECL: Enhanced chemiluminescence
HRP: Horse radish peroxidase
PGs: Prostaglandin synthesis
CYP51: Cytochrome P450 lanosterol 14a-demethylase
StAR: Steroid acute regulator protein
ER: Estrogen receptor.
